# Magnetism of Amorphous and Nano-Crystallized Dc-Sputter-Deposited MgO Thin Films

**DOI:** 10.3390/nano3030486

**Published:** 2013-08-07

**Authors:** Sreekanth K. Mahadeva, Jincheng Fan, Anis Biswas, K.S. Sreelatha, Lyubov Belova, K.V. Rao

**Affiliations:** 1 Department of Materials Science, Tmfy-MSE, The Royal Institute of Technology, Stockholm SE100 44, Sweden; E-Mails: skm@kth.se (S.K.M.); fanjincheng2006@yahoo.com.cn (J.F.); biswas.anis@gmail.com (A.B.); lyuba@kth.se (L.B.); 2 Department of Physics, Amrita Vishwa Vidyapeetham University, Amritapuri Campus, Kollam, Kerala 690525, India; E-Mail: kssreelatha@yahoo.com; 3 School of Materials and Engineering, Anhui University of Technology, Maanshan 243002, China; 4 Department of Physics, University of South Florida, Tampa, FL 33620, USA; 5 Department of Physics, Government College, Kottayam, Kerala 686013, India

**Keywords:** room temperature ferromagnetism, Mg vacancy, magnetron sputtering, O_2_ content, annealing

## Abstract

We report a systematic study of room-temperature ferromagnetism (RTFM) in pristine MgO thin films in their amorphous and nano-crystalline states. The as deposited dc-sputtered films of pristine MgO on Si substrates using a metallic Mg target in an O_2_ containing working gas atmosphere of (N_2_ + O_2_) are found to be X-ray amorphous. All these films obtained with oxygen partial pressure (P_O2_) ~10% to 80% while maintaining the same total pressure of the working gas are found to be ferromagnetic at room temperature. The room temperature saturation magnetization (M_S_) value of 2.68 emu/cm^3^ obtained for the MgO film deposited in P_O2_ of 10% increases to 9.62 emu/cm^3^ for film deposited at P_O2_ of 40%. However, the M_S_ values decrease steadily for further increase of oxygen partial pressure during deposition. On thermal annealing at temperatures in the range 600 to 800 °C, the films become nanocrystalline and as the crystallite size grows with longer annealing times and higher temperature, M_S_ decreases. Our study clearly points out that it is possible to tailor the magnetic properties of thin films of MgO. The room temperature ferromagnetism in MgO films is attributed to the presence of Mg cation vacancies.

## 1. Introduction

Magnesium Oxide with rocksalt-structure has been extensively investigated due to its exceptional properties, such as chemical inertness, high electrical resistivity, optical transparency, and low thermal conductivity [1,2,3,4]. MgO have been used widely in technology and industry, such as an insulating layer in magnetic tunnel junctions, insulating coating of electrodes in magneto hydrodynamic devices, electrodes in plasma technology, Josephson junctions and catalysis [5,6,7,8,9,10,11]. Currently, the role of defective sites in MgO films is a subject of intense research. Such defective sites, present in MgO, can introduce new electronic states, resulting in several intriguing optical, electronic and magnetic phenomena [12,13]. For example, room temperature ferromagnetism (RTFM) can arise in a MgO thin film due to the presence of point defects, which opens new possibilities for spin photonic device application [14,15]. In an earlier study, Ferrari *et al*. investigated the magnetic properties of MgO, extensively and suggested that magnetic moment appeared in the local environment of low coordinated O atoms at the surface [16]. It has also been pointed out that presence of nitrogen can influence magnetic property of MgO [17,18]. Although there are many theoretical and experimental studies regarding RTFM in MgO, there is still a lack of understanding about the origin of such phenomenon [19,20,21,22].

The main objective of the present study is to obtain a detailed insight into the role of defects and processing parameters on RTFM in MgO. In this regard, we have investigated the relation between the RTFM, the crystallinity, and Mg vacancies in the MgO films, by combining the magnetic measurements with the structural and composition analyses as a function of thermal treatment for different periods of time. Our comprehensive study reveals that the defects related to Mg-vacancies in the films are mainly responsible for RTFM in the materials.

## 2. Results and Discussion

### 2.1. Structural Properties of Films

**Figure 1:**
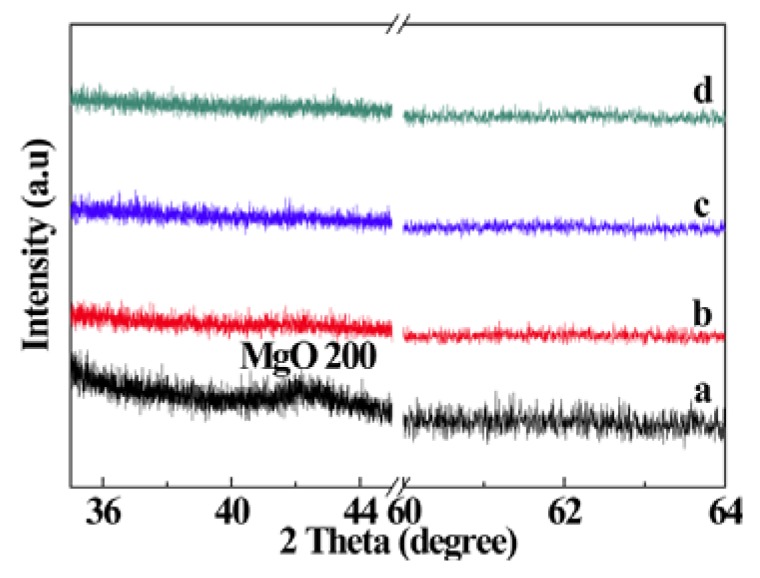
X-ray diffraction (XRD) patterns of MgO films deposited in different oxygen partial pressures: (**a**) 10%, (**b**) 20%, (**c**) 40%, and (**d**) 80%.

**Figure 2:**
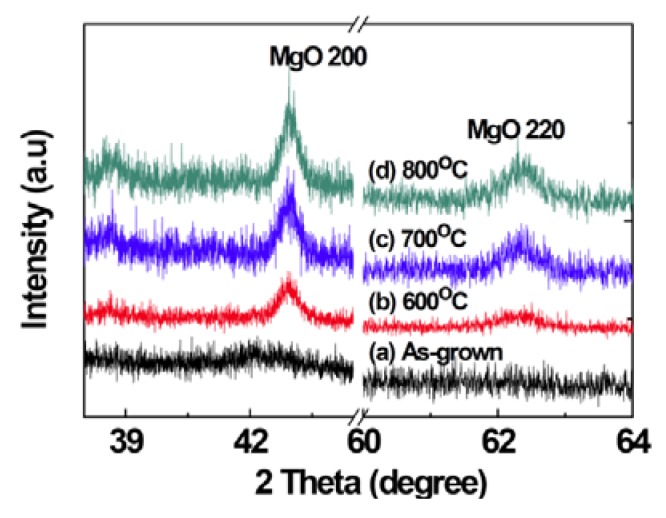
The XRD patterns of the (**a**) as-grown film deposited at P_O2_ of 10%, annealed for 1 h at (**b**) 600 °C, (**c**) 700 °C, (**d**) 800 °C respectively.

**Figure 3:**
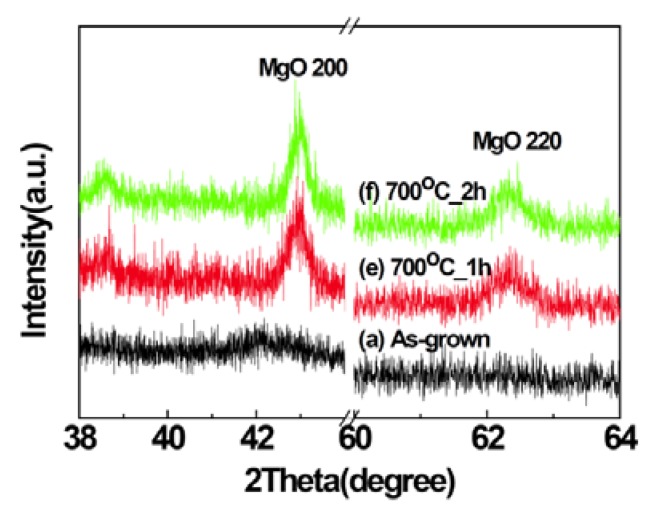
The XRD patterns of the (**a**) as-grown film deposited at P_O2_ of 10% annealed at 700 °C for (**e**) 1 h, (**f**) 2 h respectively.

**Figure 4:**
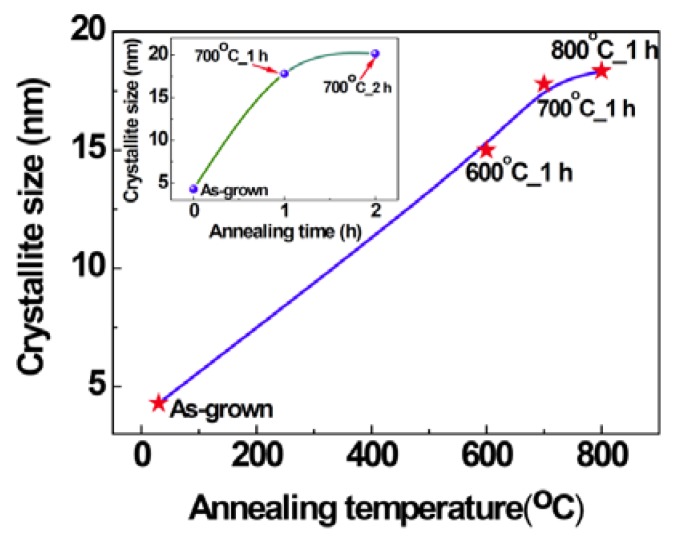
The variation of grain size with 1h anneals at various temperatures (inset: the variation of grain size with annealing time).

Figure 1 shows the X-ray diffraction (XRD) patterns of MgO films deposited with different P_O2_ in the working gas. Peaks related to MgO were not observed in the XRD patterns of the samples indicating that the films are mostly amorphous in the as-grown state. Large lattice mismatch (22.4%) between MgO (0.4213 nm) and Si (0.543 nm) and a large difference in thermal expansion coefficients (MgO: 13.5 × 10^−6^/°C, Si: 4.0 × 10^−6^/°C) hinder the possible epitaxial growth of MgO on Si substrates [23,24]. The film deposited with the P_O2_ of 10% shows a broad peak at about 42°. However, the peaks at about 42.9° and 62.3°, corresponding to (200) and (220) of MgO, respectively, are clearly visible in XRD patterns of thermally annealed samples (Figure 2, Figure 3), indicating the development of crystallinity after annealing treatment. Such a development of crystallinity can be attributed to the increasing mobility of the atoms caused by annealing [2]. The average crystallite size for the samples was estimated by using Scherrer’s formula from (200) peaks and is found to be 4.3 nm for the as grown film, deposited at O_2_ partial pressure of 10% and after the thermal treatment the grain size increases up to ~20 nm. The variation of grain size with annealing temperature and time is shown in the Figure 4. It should be noted, the films prepared in other oxygen partial pressure are predominantly amorphous in nature (Figure 1). There exists no visible peak in XRD pattern of those films. Therefore, it is not possible to evaluate grain size using Scherrer’s formula from XRD data for those films. The X-ray amorphous nature of this film may be indication of very small grain size of the films.

### 2.2. Scanning Electron Microscopy (SEM)/Focused Ion Beam (FIB) and Energy Dispersive X-Ray Analyses

**Figure 5:**
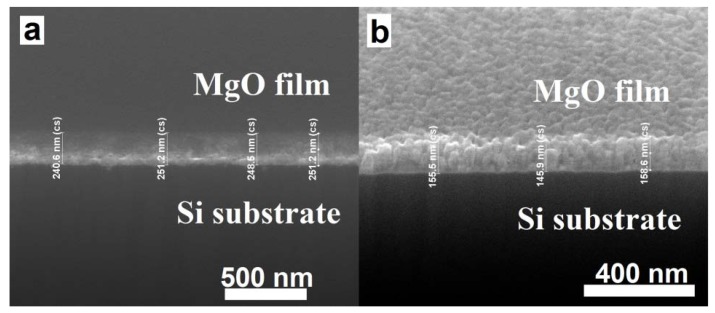
A typical cross-section of MgO films, obtained using Focused Ion Beam (FIB), in their (**a**) as-grown, and (**b**) annealed at 800 °C in air for 1 h states.

**Figure 6:**
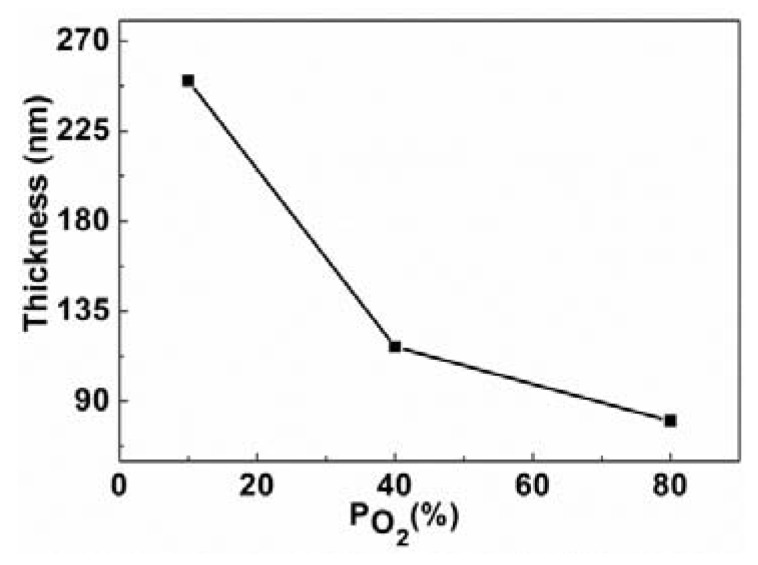
The film thickness as a function of oxygen partial pressure (P_O2_).

**Figure 7:**
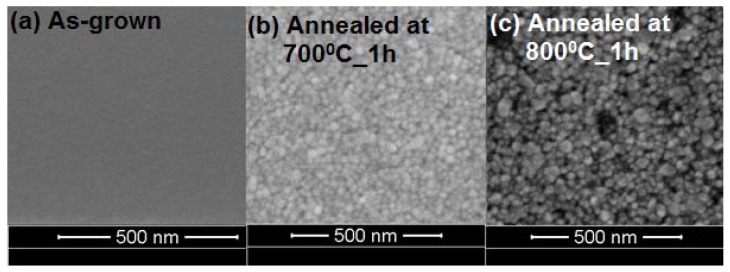
Scanning Electron Microscopy (SEM) images of (**a**) as-grown, and annealed films at (**b**) 700 °C, and (**c**) 800 °C for 1 h respectively.

The cross-section of MgO films were analyzed by FIB, the Figure 5 shows the typical cross-section of as-grown and annealed (800 °C) MgO films and the thickness of the films found to be in the range of ~80–250 nm. Figure 6 shows the film thickness as function of P_O2_. It is clear that the film thickness decreases with the increase of P_O2_. In the sputtering process, plasma of non-reactive ions is created by a potential difference inside a vacuum chamber, which falls on the target material and breaks the atoms and are then deposited on the substrate. When the P_O2_ increases the formation of the number of non-reactive ions decreases and consequently results in the decrease of the film thickness. It can be seen that the as-grown films were amorphous and after annealing, the structure of the films was improved and the well-shaped uniform grains were formed, which is consistent with XRD results (Figure 2). Typical SEM images of the films are shown in Figure 7, which clearly indicates the increase of grain size with increase of annealing temperature. Also note that the thickness of the films decreased after annealing in our experiments, suggesting that the films become denser and probably contain fewer defects.

**Figure 8:**
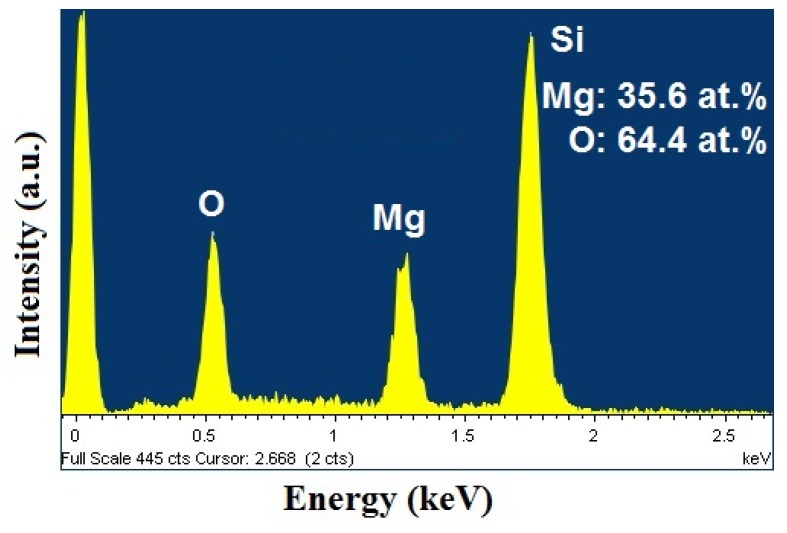
The energy dispersive spectroscopic (EDS) spectrum for MgO film deposited with O_2_ content of 10% in the working gas.

Energy dispersive spectroscopic (EDS) was used to analyze the compositions of MgO films. Figure 8 shows the typical EDS spectrum of MgO film deposited with the O_2_ content of 10%. Only Si, Mg, and O peaks were detected, indicating the absence of any form of transition metal contamination in the films within the limits of detection. Obviously, the observed peak of Si should be ascribed to the substrates. The inset of Figure 8 shows the corresponding atomic % of Mg, and O. The ratio of O to Mg was ~1.8 (>1), which suggests that there may be a lot of deficiency Mg vacancies in the MgO films.

### 2.3. Magnetic Properties

**Figure 9:**
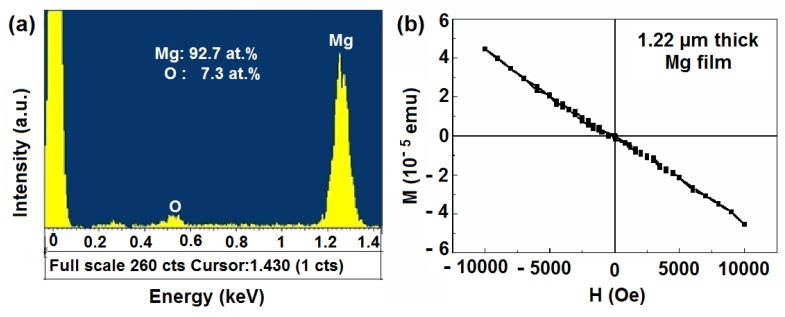
The (**a**) EDS spectrum and (**b**) M-H loop for the 1.22 µm thick Mg film.

Prior to the magnetic measurement of the thin films, to rule out the possibility of the magnetic contamination in the Mg target, we have investigated a thick film (~1.2 μm) of the target by using Energy Dispersive X-ray spectroscopy to confirm the absence of any impurity and also studied M-H loops of the thick film. None of these studies exhibited any possible existence of even a signature of ferromagnetism. Energy Dispersive X-ray spectroscopy study reveals presence of Mg (92.7%) and O (7.3%) in this film. Thus, the ferromagnetic property of the films is intrinsic. Both the EDS and SQUID data of the thick Mg film (~1.2 μm) of the target are presented in the Figure 9a,b, respectively.

One may doubt whether such a large M_S_ value is the property of MgO films or it arises from small amount of magnetic contamination present in the sample. To rule out such a possibility, the target as well as the substrates were checked by EDS analyses which only confirmed the absence of any impurity. We also studied M-H curve of the parts of targets and substrates and found no evidence of possible ferromagnetism. Thus, the ferromagnetic property we observe in the thinner films is intrinsic.

**Figure 10:**
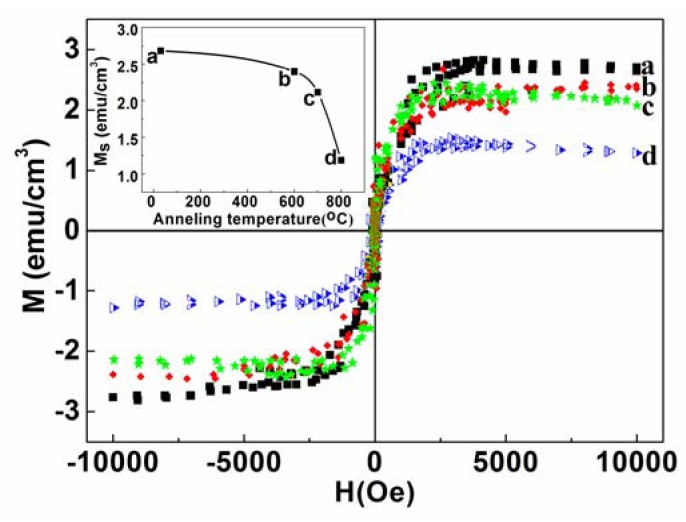
Room-temperature M-H loops for the as-grown (**a**), and annealed MgO films in air for 1 h at (**b**) 600 °C, (**c**) 700 °C, and (**d**) 800 °C, respectively. The (M-H) loops are shown after correcting for the diamagnetic contribution from Si-substrate. The Inset shows the M_S_ values at room-temperature (RT) observed as a function of annealing temperature.

**Figure 11:**
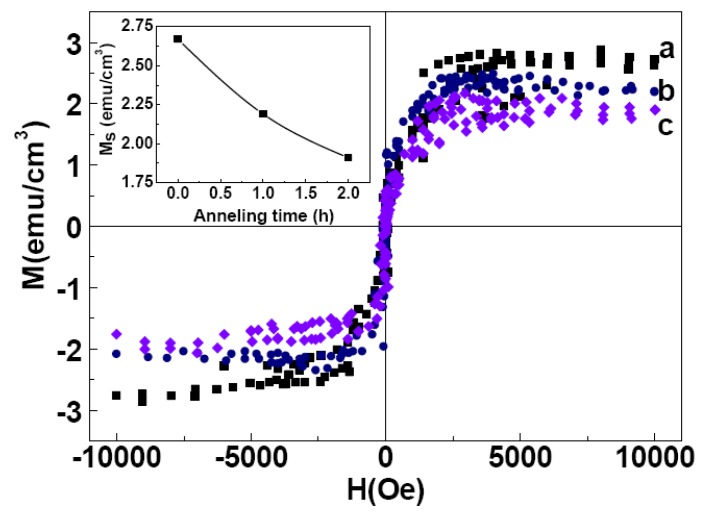
The room temperature M-H of the as-grown (**a**), and annealed MgO Films at 700 °C for 1 h (**b**) and 2 h (**c**) respectively. The (M-H) loops are shown after correcting for the diamagnetic contribution from Si-substrate. Inset shows the M_S_ values as a function of annealing time.

We have studied the effect of thermal annealing on the magnetic properties of MgO films, systematically. The film, deposited at 10% oxygen partial pressure, was chosen for this purpose as it exhibits some signature of (200)-peak in its XRD pattern. Thermal treatment was performed for the sample deposited in O_2_ content of 10% at 600, 700 and 800 °C respectively in air for 1 h. RTFM was observed in the as-grown and annealed MgO films, which is consistent with previous reports of magnetism in undoped oxides, such as MgO, ZnO, TiO_2_, In_2_O_3_ and HfO_2_ [19,25,26,27,28]. Figure 10, Figure 11 show the magnetic field dependence of magnetization (M(H)) curves at room temperature for as-grown and annealed films (deposited at P_O2_ ~10%) after carrying out the standard diamagnetic corrections for the substrate effects. In a recent study, Gao *et al*. considered that V_Mg_ could introduce strong ferromagnetism in MgO films and the ferromagnetism depended on the V_Mg_ concentration in the MgO films [19].

Annealing resulted in a reduction in the M_S_ value from ~2.69 to 1.18 emu/cm^3^ (inset, Figure 10). The decrease could tentatively be ascribed to the reduction of V_Mg_ in MgO films due to annealing. The contribution of one V_Mg_ in the total magnetic moment is 1.9 μB for MgO [19]. Therefore, the decrease in the magnetization of 1.5 emu/cm^3^ actually corresponds to a reduced number of V_Mg_ of 8.5 × 10^19^/cm^3^ in the annealed MgO films. A similar phenomenon was also observed for MgO films annealed at 700 °C for different times (Figure 11) resulting in a reduction of the M_S_ value of the sample from ~2.69 to 1.9 emu/cm^3^. Inset shows the M_S_ values as a function of annealing time.

By investigating the XRD patterns of the MgO films before and after annealing, it can be inferred that the crystallinity grows in the films due to the annealing in air, as shown in the Figure 2, Figure 3. Consequently, the sharp decrease of M_S_ at room temperature for the sample after annealing could be attributed to the increased crystallinity. On the other hand, this result confirms that magnetism in those MgO films is surely intrinsic rather than from any other transition metal contaminations [29]. The atmosphere during annealing may not only produce cation vacancies but also improve the crystallinity of the samples.

**Figure 12:**
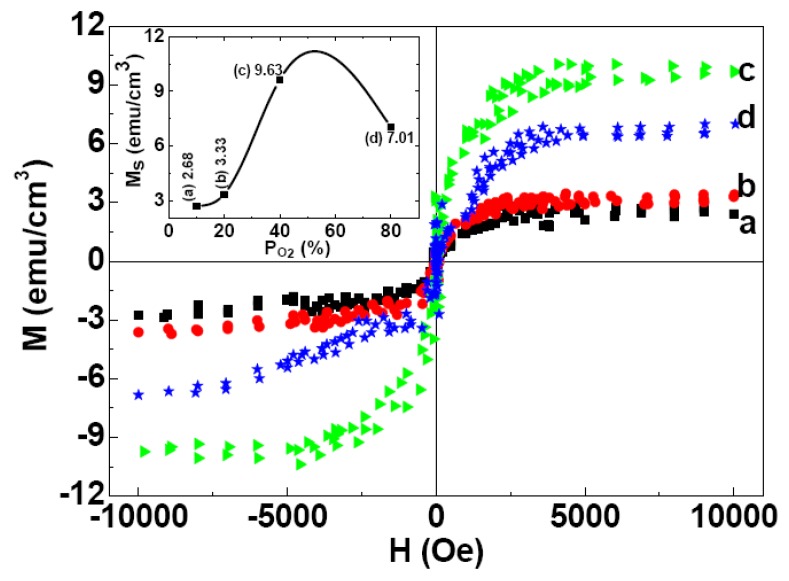
The room temperature (M-H) loops for as-deposited MgO films with different O_2_ contents in the working gas: (**a**) 10%, (**b**) 20%, (**c**) 40%, (**d**) 80%. The loops are shown after correcting for the diamagnetic contribution from Si-substrate. Inset: dependence of room temperature M_S_ values for films deposited under different P_O2_ in the working gas.

In order to understand the origin of the ferromagnetism (FM) observed in MgO films more clearly we also investigated the effect of oxygen partial pressure during deposition on the magnetization of MgO films at room temperature (RT). Figure 12 shows the M-H loops measured at room temperature for MgO films deposited with the various P_O2_ in working gas. Inset of the Figure 12 shows M_S_ as a function of P_O2_ in working gas. The results indicates that P_O2_ in working gas plays an important role to determine the magnetic properties of MgO films and ferromagnetism may originate from the defects at the cation sites, which is consistent with the theoretical results [19,30]. Initially, the increase in O_2_ content of the working gas increases the ratio of O/Mg (>1), which could result in the enrichment of V_Mg_ in the films and consequently the enhancement of M_S_ is observed. However, further increase of O_2_ content may form O-related defects, such as oxygen interstitial (O_i_) and oxygen antisite (O_Mg_). These defects would break up the long range ferromagnetic chains and lead to lower net magnetization in the films.

**Figure 13:**
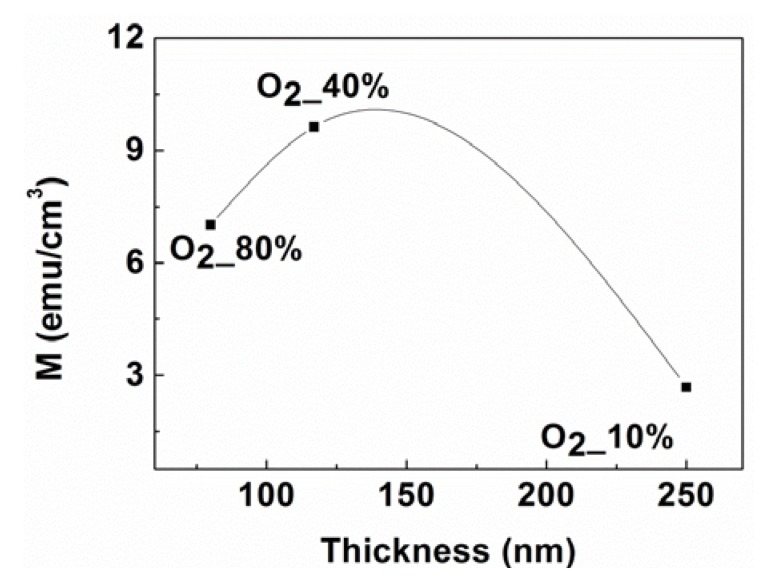
The M_S_ value as a function of film thickness for as-deposited MgO films in an ambience of different O_2_ contents.

Figure 13 shows the M_S_ values observed as a function of film thickness for as-deposited MgO films with different O_2_ contents. The M_S_ of the films increases with the film’s thickness and then decreases, which may be due to the increase in V_Mg_ concentration at first and then due to the decrease of this concentration when the defects coalescence in thicker films due to the changes in the separation distance between the defects and also because of the decreasing strain as films become thicker in the case of crystalline films.

It should be noted that the obtained M_S_ value for the films was low at lower oxygen content, indicating that the ferromagnetism does not arise from oxygen vacancy. Hence, the ferromagnetic ordering arises primarily due to the presence of cation vacancy, (V_Mg_). Obviously the amorphous oxides contains more defects in comparison with its crystalline counterpart. Since the RTFM is defect induced, it must be enhanced when the oxides are in the amorphous state as observed in the present case. Thus, the oxygen content in sputtering and annealing conditions are important factors in determining the RTFM in oxide thin films. These two factors are competitive and, in turn determine the nature and concentration of defects. Photoluminescence, electron nuclear double resonance, or positron annihilation techniques may be also useful to understand role of defects on RTFM in these films.

## 3. Experiment

### 3.1. Fabrication of MgO Thin Films

MgO films were deposited on Si substrates at room temperature by reactive DC magnetron sputtering using 99.99% pure Mg metallic target in a well-controlled (O_2_ + N_2_) atmosphere. The Si substrates were etched with hydro fluoric acid (HF) to remove surface native oxide (SiO_2_), and then were cleaned in the ultrasonic baths of acetone, isopropanol and de-ionized water, and blown dried in nitrogen. The vacuum chamber was evacuated to a base of 10^−3^ Pa. Prior to the films’ growth, the Mg target was cleaned by sputtering it for 30 min. (N_2_ + O_2_) maintained at 0.15 Pa was used as the working gas. N_2_ and O_2_ were introduced into the chamber via two digital mass flow meters. During deposition, the oxygen partial pressure (P_O2_ = oxygen pressure/total pressure of oxygen and nitrogen) was kept at 10%, 20%, 40%, and 80% of the total pressure, respectively. In order get an appropriate film thickness of the MgO films in this study were deposited for three hours with the DC power of ~55 W. Thermal treatment performed in air for the sample deposited with the (P_O2_) 10% at 600, 700 and 800 °C respectively for 1 h and 700 °C for 2 h.

### 3.2. Characterization Techniques

The structure of MgO films was characterized by X-ray diffraction (XRD; Siemens D5000, Siemens, Munich, Germany) with Cu Kα (λ = 1.5405 Å) radiation. The thickness of the films was determined from the cross-section analysis by a Dual-Beam UHR SEM/FIB system (FEI Nova 600 Nanolab; NanoPort, Eindhoven, The Netherlands). Energy dispersive spectroscopic (EDS, Oxford Instruments, Abingdon, UK) analyses were carried out to investigate the compositions of the films. The magnetic properties of MgO films were measured at room temperature by using a commercial SQUID magnetometer (Quantum Design Inc., San Diego, CA, USA).

## 4. Conclusions

MgO films were grown on Si substrates by direct current (DC) sputtering from a metallic Mg target with various O_2_ contents in working gas, and the effects of thermal annealing on the structural and magnetic properties were investigated. Our results reveal that there exists a close correlation between the room temperature ferromagnetism, the crystallinity, and the magnesium vacancy concentrations in the MgO thin films. All as-grown films were found to be amorphous most likely due to the large lattice mismatch between MgO and Si substrate. After annealing, the films became crystalline and the M_S_ values decreased. The origin of the RTFM could be attributed to the Mg cation vacancies. The P_O2_ in working gas plays an important role to determine the M_S_ value of as-grown MgO films. When P_O2_ increased from 10% to 80%, M_S_ values initially increased up to P_O2_ of 40% and then decreased at higher oxygen content in the working gas. EDS results show that the ratio of O/Mg in MgO films is greater than 1, indicating the presence of high density of Mg vacancies in MgO films. Furthermore, we have found that less crystallinity can increase the ferromagnetism of MgO thin films due to larger amounts of Mg vacancies. Our results not only demonstrate an important approach to obtain room temperature ferromagnetic MgO films but also help to understand the origin of ferromagnetism in MgO films.
